# Effect of standard phlebotomy on myocardial and hepatic iron levels in newly diagnosed cardiac asymptomatic hereditary hemochromatosis subjects with C282Y homozygosity

**DOI:** 10.1002/jha2.662

**Published:** 2023-02-23

**Authors:** Yukitaka Shizukuda, W. Patricia Bandettini, Douglas R. Rosing

**Affiliations:** ^1^ Cardiovascular Branch National Heart Lung and Blood Institute National Institutes of Health Bethesda Maryland USA; ^2^ Division of Cardiovascular Health and Disease Department of Internal Medicine University of Cincinnati Ohio USA; ^3^ Division of Cardiology Department of Internal Medicine Cincinnati VA Medical Center Cincinnati Ohio USA

**Keywords:** hepatic iron, hereditary hemochromatosis, MRI, myocardial iron, phlebotomy

1

Standard phlebotomy effectively reduces systemic and tissue iron overload (IO) in hemochromatosis patients [1, 2, 3]. However, the long‐term therapeutic effect of phlebotomy on tissue IO in newly diagnosed cardiac asymptomatic hereditary hemochromatosis (HH) subjects during the course of standard therapy in a prospective cohort is not described. Up to now, only one cohort study examined changes in myocardial iron levels after phlebotomy therapy in newly diagnosed HH subjects as far as we know [4]. The study showed myocardial iron levels did not change significantly after 3 months of the standard therapy in the newly diagnosed HH subjects; however, it lacked longer term follow‐up, was limited to myocardial iron, and had no control subjects. Therefore, we have examined the effect of standard phlebotomy therapy on myocardial and hepatic iron content measured with T2* (T2 star magnetic vector decay constant) derived from noninvasive magnetic resonance imaging (MRI) in our cohort of cardiac asymptomatic HH subjects (*n* = 22) with C282Y homozygosity as well as in age‐gender matched normal volunteers (NV, *n* = 21) who lacked the HFE mutation to cause HH over a 5‐year follow‐up in the National, Heart, Lung, and Blood Institute (NHLBI)‐sponsored “Heart Study of Hemochromatosis” (ClinicalTrials.gov number, NCT00068159) [5, 6, 7].

The study was approved by the Institutional Review Board of the NHLBI of the National Institutes of Health (NIH) and was performed in accordance with the ethical standards as laid down in the Declaration of Helsinki and its later amendments or comparable ethical standards [8]. Consent for the study was obtained from the participants. The demographic data of these groups were published in our previous articles [5, 7]. The average age of the newly diagnosed HH group was 48 ± 11 years (data are mean ± SD, 27% female) and that of the NV group was 48 ± 8 years at baseline (33% female). They showed no significant left ventricular systolic dysfunction as compared to NV subjects [5, 7]. The research nurses for this study communicated with the HH subjects throughout the study period to ensure the maintenance of the standard phlebotomy therapy by their local clinical care providers.

Myocardial and liver T2* values and blood test results were obtained from the clinical information system of the NIH Clinical Center. T2* of myocardium and liver was measured from the images obtained with a 1.5 T MRI scanner using a gradient echo sequence [9, 10]. Smaller T2* measurements represent higher tissue iron level. MRIs were performed at baseline, 6‐month, and 5‐year follow‐ups in HH subjects and baseline and 5‐year follow‐up in NV subjects. For data analysis, the normality test of each parameter was conducted with the Shapiro‐Wilk test. If the normality of the data was established, an unpaired *t*‐test was used to compare the HH and NV groups at each time point and to compare measurements between baseline and 5‐year follow‐up in the NV group. ANOVA with post hoc Tukey‐Kramer HSD tests was used to compare different time points in the HH group. If the normality of the data was not established, nonparametric statistical methods were used instead. The Wilcoxon test was used instead of the unpaired *t*‐test, and Kruskal–Wallis test with post hoc Wilcoxon tests was used instead of ANOVA with post hoc Tukey‐Kramer HSD tests to analyze the data whose normality were not established. The χ‐square test was used to compare the frequency of female sex between the groups and time points in the same group. *p*‐Values less than 0.05 were considered to be statistically significant.

At baseline, 20 HH and 20 NV subjects had MRI and both myocardial and hepatic T2* could be obtained in 17 HH subjects and 20 NV subjects. At 6‐month follow‐up, 17 HH subjects had MRI with evaluable T2* in all cases. At 5‐year follow‐up, 12 HH and 18 NV subjects had MRI with evaluable T2* in all cases.

Among newly diagnosed HH subjects whose tissue T2*were evaluable, the measures of systemic IO as well as alanine aminotransferase and aspartate aminotransferase were significantly improved over time by standard phlebotomy therapy (Table 1).

**TABLE 1:** Clinical characteristics of subjects with evaluable MRI T2* measurements in newly diagnosed hereditary hemochromatosis and age‐sex matched normal volunteer cohorts over 5 years.

	BL		6 M	5Y	
	HH	NV	HH	HH	NV
Variables	(*n* = 17)	(*n* = 20)	(*n* = 17)	(*n* = 12)	(*n* = 18)
Age (years)	48 ± 9	49 ± 8	49 ± 10	54 ± 12	55 ± 8*
Female sex (% female)	4 (24%)	7 (35%)	3 (18%)	4 (33%)	6 (33%)
Ferritin (μg/L)^#^	1102 ± 839^†^	97 ± 77	305 ± 500*	67 ± 38*	96 ± 73
Transferrin sat (%)	76 ± 19^†^	25 ± 10	50 ± 29*	60 ± 20^†^	26 ± 11
Hemoglobin (g/dL)	15.0 ± 1.2	14.2 ± 1.2	13.9 ± 0.9*	14.9 ± 1.4	14.4 ± 0.9
Hematocrit (%)	43.6 ± 3.4	42.0 ± 3.7	40.6 ± 2.6*	43.5 ± 3.7	43.3 ± 2.9
ALT (IU/mL)	48 ± 21^†^	28 ± 13	29 ± 17*	35 ± 12	34 ± 11
AST (IU/mL)	34 ± 8	25 ± 6	25 ± 6*	18 ± 4*	20 ± 7
Glucose (mg/dL)	97 ± 15	99 ± 12	95 ± 21	97 ± 12	98 ± 11
Creatinine (mg/dL)	0.9 ± 0.1	1.0 ± 0.2	0.9 ± 0.1	0.8 ± 0.3	0.9 ± 0.2

Abbreviations: 5Y: 5‐year follow up; 6 M: 6‐month follow up; ALT: alanine aminotransferase; AST: aspartate aminotransferase. See Methods for statistical analyses.; BL: baseline; Hct: hematocrit; Hgb: hemoglobin; HH: newly diagnosed subjects with hereditary hemochromatosis homozygous for C282Y; MRI: magnetic resonance imaging; NV: age‐gender matched normal volunteers; sat: saturation

* *p* < 0.05 versus BL.

^†^ *p* < 0.05 versus NV. Data are expressed as mean ± SD.

^#^ The data are not normality distributed.

The newly diagnosed cardiac asymptomatic HH subjects did not show significantly elevated cardiac iron levels at baseline compared to NV subjects (T2*: 31.3 ± 7.0 ms vs. 34.1 ± 7.6 ms, *p* = 0.123), and this finding persisted at the 5‐year follow‐up time point (T2*: 28.0 ± 7.5 ms vs. 31.2 ± 12.2 ms, *p* = 0.408, Figure 1). The hepatic iron level was elevated at baseline compared to the NV group (T2*: 8.1 ± 6.7 ms vs. 26.8 ± 4.7 ms, *p* < 0.01, Figure 1). However, this difference disappeared at the 5‐year follow‐up (T2*: 28.1 ± 6.4 ms vs. 28.6 ± 6.3 ms, *p* < 0.966, Figure 1). Myocardial and hepatic T2* of HH subjects at 6‐month follow‐up were 32.5 ± 7.3 and 11.3 ± 7.7 ms, respectively (Figure 1).

**FIGURE 1:**
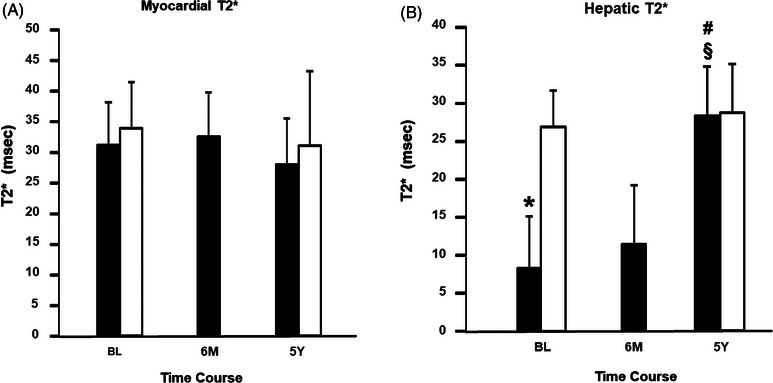
Tissue iron level measured with T2* using magnetic resonance imaging of myocardium (panel A) and liver (panel B) are shown. Black bars denote newly diagnosed hereditary hemochromatosis (HH) cohort subjects with C282Y homozygosity. White bars denote the age‐sex‐matched normal volunteer (NV) cohort subjects who lacked HFE and other mutations causing HH. BL = baseline, 6 M = 6 months, 5Y = 5 years, * = *p* < 0.05 with Wilcoxon test versus NV, § = *p* < 0.05 versus BL with Kruskal–Wallis test with post hoc Wilcoxon tests, # = *p* < 0.05 versus 6 M with Kruskal–Wallis test with post hoc Wilcoxon tests.

This study is the first to systematically show that both myocardial and hepatic IO were prevented or improved in the newly diagnosed HH cohort subjects with standard phlebotomy. The myocardial iron levels of HH subjects were comparable to age‐gender matched NV subjects throughout the study period of 5 years. The hepatic iron level was elevated at baseline in the HH population; however, it normalized to the level of NV subjects after 5 years of conventional phlebotomy therapy. We have reported persistently elevated oxidative stress levels [11] in this population despite their normal myocardial tissue iron levels while receiving standard phlebotomy therapy [6, 12] and speculated that the elevated oxidative level may potentially be responsible for long‐term tissue damage related to HH [11, 13, 14]. Thus, there still remains a concern for long‐term tissue damage in the HH population.

This study has several limitations including the small cohort size, the use of only MRI to measure tissue iron levels, and being limited to HH with C283Y homozygosity. In addition, the standard of phlebotomy therapy might vary locally; however, our blood testing results assure that HH subjects were consistent with the accepted therapeutic effect of maintenance standard phlebotomy therapy [3, 15].

In conclusion, our prospective study demonstrated that conventional phlebotomy therapy over 5 years was effective in preventing both myocardial and hepatic iron accumulation when it was initiated at the cardiac asymptomatic stage of HH subjects with C282Y homozygosity. Further investigations are warranted to generalize this observation to a wider range of hemochromatosis populations.

## AUTHOR CONTRIBUTIONS

Dr. Yukitaka Shizukuda designed the study, recruited the subjects, collected data, performed data analysis, performed the literature search, and drafted this article. Dr. Patricia Bandettini collected data and critically edited the work. Dr. Douglas Rosing designed the study, drafted this article, and critically edited the work.

## CONFLICT OF INTEREST STATEMENT

The authors have no conflict of interest to disclose.

## FUNDING INFORMATION

National Heart, Lung, and Blood Institute of the National Institutes of Health

## ETHICS STATEMENT

The study was approved by the Institutional Review Board of the NHLBI of the National Institutes of Health (NIH) and was performed in accordance with the ethical standards as laid down in the Declaration of Helsinki and its later amendments or comparable ethical standards. Consent for the study was obtained from the participants.

## Data Availability

The data that support the findings of this study are governed by the National Heart, Lung, and Blood Institute (NHLBI) of the National Institutes of Health. This study protocol does not have the approved data sharing plan by the NHLBI. However, a data sharing request may be submitted to the NHLBI for consideration on a case‐by‐case basis.
